# Supplementary material for: Magnetisation switching of FePt nanoparticle recording medium by femtosecond laser pulses
Source: Sci Rep. 2017 Jun 23;7:4114. doi: 10.1038/s41598-017-04167-w (PMC5482900; doi:10.1038/s41598-017-04167-w)
Supplement: Supplementary file 1 — Supplementary information [file 41598_2017_4167_MOESM1_ESM.pdf]

Supplementary Information

Magnetisation switching of FePt nanoparticle recording medium by femtosecond laser pulses

R. John[1], M. Berritta[2], D. Hinzke[3], C. Müller[4], T. Santos[5], H. Ulrichs[6], P. Nieves[7,8], J. Walowski[1], R. Mondal[2], O. Chubykalo-Fesenko[7], J. McCord[4], P. M. Oppeneer[2], U. Nowak[3], M. Münzenberg[1]

[1] Department of Physics, Ernst-Moritz-Arndt-University, 17489 Greifswald, Greifswald, Germany.
[2] Department of Physics and Astronomy, Uppsala University, P.O. Box 516, SE-75120, Uppsala, Sweden.
[3] Department of Physics, University of Konstanz, 78457 Konstanz, Germany.
[4] Institute for Materials Science, Kiel University, 24143 Kiel, Germany.
[5] Western Digital Corporation, San Jose, CA 95131, USA.
[6] I. Phys. Institut, Georg-August-University, 37077 Göttingen, Germany.
[7] Instituto de Ciencia de Materiales de Madrid, CSIC, Cantoblanco, 28049 Madrid, Spain.
[8] International Research Centre in Critical Raw Materials for Advanced Industrial Technologies, ICCRAM, Universidad de Burgos, 09001 Burgos, Spain.

1. Additional MOKE data for the saturated starting condition

The FePt recording media was prepared in two states before the all-optical laser writing experiments. One is the demagnetised condition with close to 50% 'up'/ 50% 'down' magnetised magnetic grains, the second is the magnetised condition with close to 100% up/ 0% down magnetised grains. Because of the large anisotropy fields of the magnetic recording media to reach long data stability, the initial condition is long term stable. The conditioning worked as follows. In both cases, the sample was heated to 750 K well above the Curie temperature (T_c =700 K). Then, to reach the saturated state with close to ~100% 'up'/ 0% 'down' condition of the magnetisation direction, the sample was heated to 750 K and a field strength of 400 mT applied perpendicular to the sample surface during cooling. This procedure allows a direct comparison to the demagnetised state with to 50% 'up'/ 50% 'down' condition where the same procedure of heating was applied, but the sample is cooled in zero field. In addition, we tested in reference experiments with sampled magnetised in a large applied field of above >5 T without any heating that these show the same results.

The magneto-optical contrast image in Fig. 1 presents only the 50% 'up'/ 50% 'down' condition demagnetised starting condition. Fig. 1S shows the magneto-optical contrast image in the saturated ~100% 'up'/ 0% 'down' starting condition.

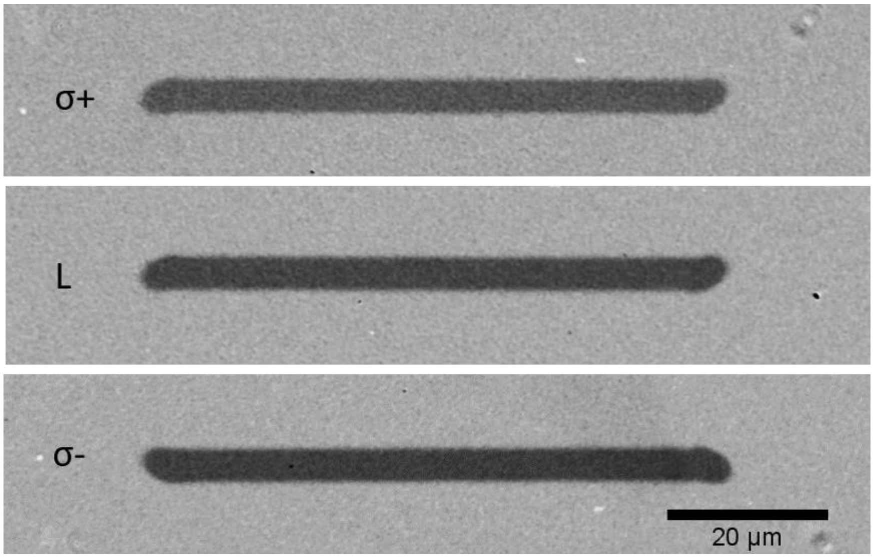

Fig. S1. Magnetisation switching with various polarisations σ+, L and σ- of the femtosecond laser pulses (labeled on individual writings) with an initial magnetisation which is saturated 100% in the perpendicular direction to the surface, showing the writing for σ- and for σ+ polarised femtosecond laser pulse beam corresponding to the line profiles shown in Fig. 1d.

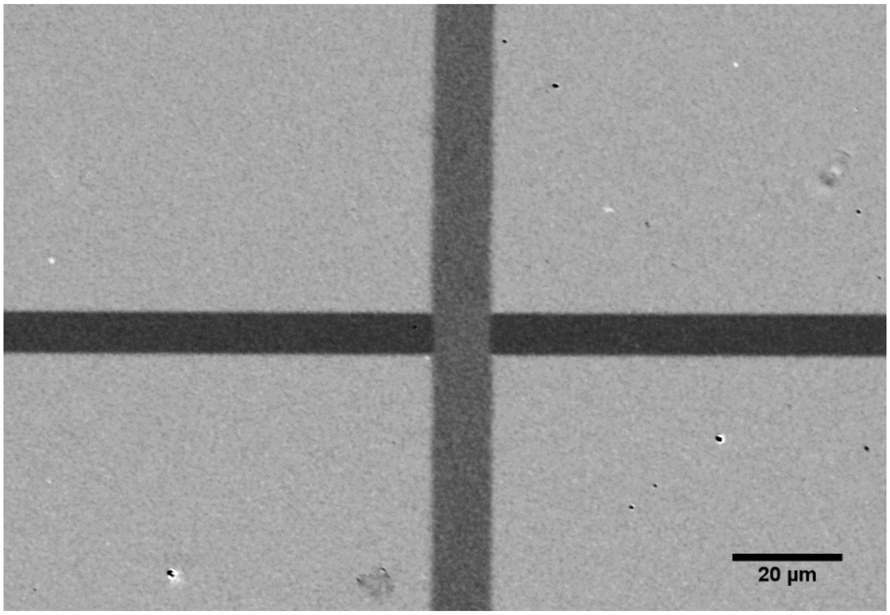

Fig. S2: Overwriting of a previously written line pattern with an initial magnetisation which is saturated 100% in the perpendicular direction to the surface, showing the writing for σ- and for σ+ polarised femtosecond laser pulse beam.

The magneto-optical contrast was enhanced by background subtraction of images with reversed magneto-optical contrast by switching between two different analyser angle settings in the microscope, follows and optimization procedure for each specific sample during imaging. In general, the contrast does not allow an absolute comparison for different measurements in between the samples. Therefore, the contrast is given as arbitrary unit. To allow the absolute comparison for different writing conditions, the settings have not been changed in between different images on one sample. The saturated state close to 100% 'up' is seen in the Fig. 1S as a light gray contrast, the lines for different writing with σ+, L and σ- polarised femtosecond laser pulses are seen as different darker lines.

The line scans shown in Fig. 1d of the main manuscript are extracted from the image data. Using the contrast for p_u = 50% (L) for the line written with linearly polarised laser pulses and p_u = 100% before the writing as a reference, the corresponding values of p_u equal 63% (σ+),and 41% (σ-) of M_S can be extracted from the line scans. The color code in the magneto-optical image is set here for p_u = 41% to black and p_u = 100% to white.

2. Magnetic characterization (P-MOKE)

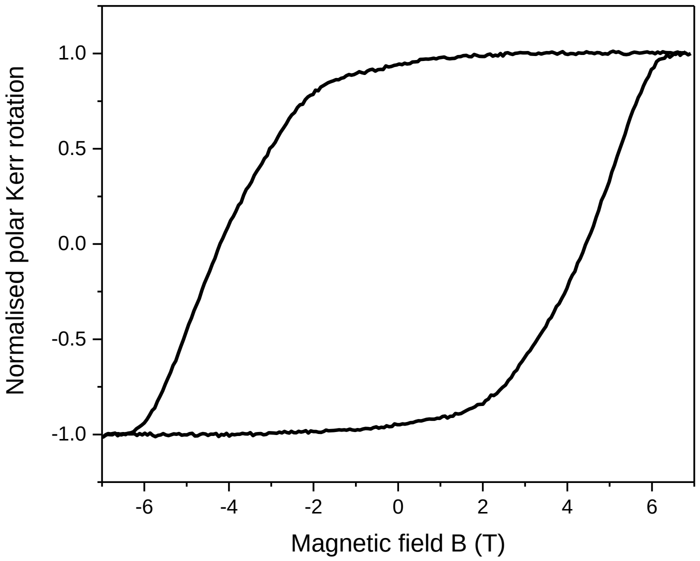

Fig. S3: Hysteresis curves for the granular recording media reveal coercive field μ_0H_C ~ 4 T, μ_0H_S~ 6 T and a remanence of 97-98% M_S. The hysteresis is measured by the magneto-optical polar Kerr effect (p-MOKE).

The FePt magnetic recording media was developed for heat assisted magnetic recording and subsequently optimized for large coercivity and small grains. A detailed study can be found in the work by D. Weller et. al. [1]. The coercivity is typically at around 4T and saturation fields of above 6T. In remanence the hysteresis shows a squareness of about 0.97-0.98 M_S, which corresponds to p_U 98.5-99% 'up' states of the magnetic grains in the remanence condition, which is close to ~100% 'up'/ 0% 'down' condition.

3. Summary of the state of art in the literature for multiple shot all-optical magnetic writing

Table 1: Comparison to works from other groups reporting multi-shot writing by femtosecond laser pulses.

**System**	**Number of shots ($n_{1/2}$)**	**Experimental Method**	**Degree of switching (max. achieved)**	**Fluence**	**Mechanism**	**Reference**
FePt nanoparticle	~4.4(3)	Kerr microscopic contrast	63% (P_{up} for σ+)<br>41% (P_{up} for σ-)	~68GW/cm^2<br>6-10 mJ/cm^2<br>(250 kHz)<br><br>30 mJ/cm2<br>(20 kHz)	Stochastic asymmetric switching, IFE and LLB	This work
Pt/Co/Pt film	~10-15	Hall bar device	80%-100%, domain formation	10 mJ/cm^2<br>(5kHz)	Demag-netization followed by a helicity-dependent accumulation by domain wall motion	El Hadri *et al.* [2]
GdFeCo alloy	~1	Hall bar device	100%, domain formation	"-"	Single pulse ultrafast switching	El Hadri *et al.* [2]
FePt, granular	~20-40	Hall bar device	75% (P_{up} for σ+)<br>25% (P_{up} for σ-)	35-80 mJ/cm^2	Rate model with Neel Brown law	Takahashi, *et al.* [3]
Co/Pt multilayers	Domain grows with each shot	Kerr microscopic contrast	100%, domain formation	0.5 mJ/cm^2<br>long 4 ps pulse<br>(500 Hz)	Helicity dependent domain wall motion	Medapalli *et al*. [4]

We give here table of the reversal capabilities achieved by multiple pulse writing by other groups. The number of shots is here given as the number of shots needed to reach half of the value of the saturation ($n_{1/2}$). Also, the writing rate after magnetisation accumulation is given for a hypothetically infinite number of shots in saturation as degree of switching for each work. The values given from other manuscripts are estimated graphically. However, these values are difficult to compare because of the slightly varying conditions that could give a large effect. This numbers depend strongly on the sample and experimental details, the sample absorption, pulse length, repetition rate etc.. These experimental parameters are also given in the table. Please note the

difference between granular media where the nanograins switch individually and films where domain formation is present.

4. Additional Kerr microscopy data for the multiple shot experiments

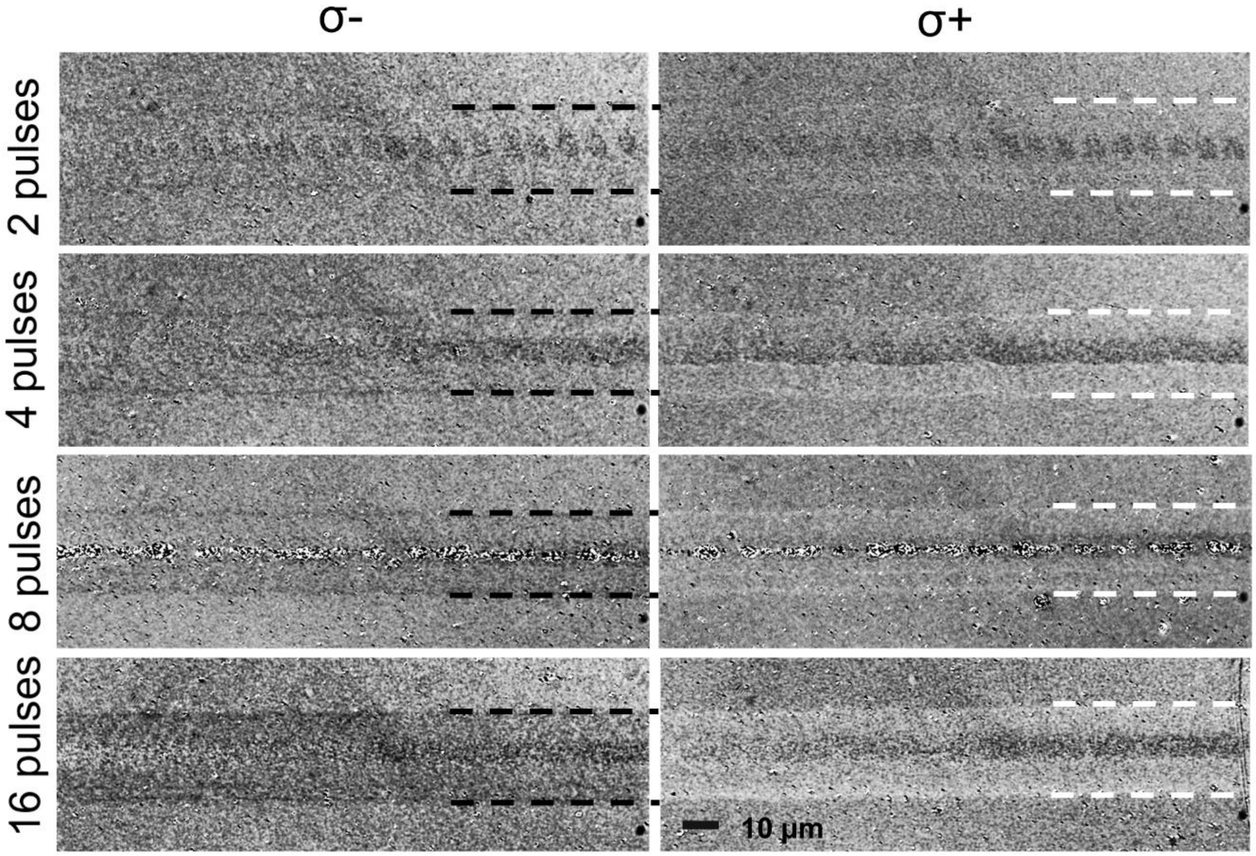

Fig. S4: Two shot to multiple shot experiments, starting from the demagnetised recording media. Saturation is reached between 15 to 120 pulses of writing. The dark centre is not of magnetic origin and shows some excess heating and a structural modification of the FePt nanoparticles, which allows us to identify the pulse train distance. The borders of the all-optical writing are marked with the dashed lines to help to identify the contrast change.

To illustrate the technical details for the two shot to multiple shot writing, we included in here the Kerr microscopy data of the writing pattern with a larger area of view. The average fluence that was used was higher as in the continuous writing experiment sweeping the laser beam given in Fig. 1 and hence the local fluence at the centre of the beam was quite high making a damage. This damage is seen as a darker well separated spot in the upper line (2 pulses). This contrast is helicity independent. It helps to identify and find the spot region. The magnetic switching is most visible towards the periphery of the beam where the fluence falls in the switching window. The borders of all-optical writing are marked with the dashed lines to help to see the contrast. Here the contrast gets slightly darker or slightly brighter, dependent of the helicity of the pump beam.

Alternative mechanism by direct angular momentum transfer

In the following we calculate an alternative mechanism of a direct transfer of the momentum of the photon. It is a very interesting direct consequence of angular momentum conservation and in fact also quite established. Already in the 1930ties Richard Beth proved, by setting a birefringent plate into rotation, that an angular of a photon of circular polarisation can be measured [5]. This is used for example in optical tweezers to date to set small particles into rotations [6]. The two questions arise, is there enough momentum in the reflected photons, e.g. how many photons are present per atom for this range of fluence, and is there a mechanism to transfer the angular momentum on a microscopic, atomic scale to the electron system and then to the spin system?

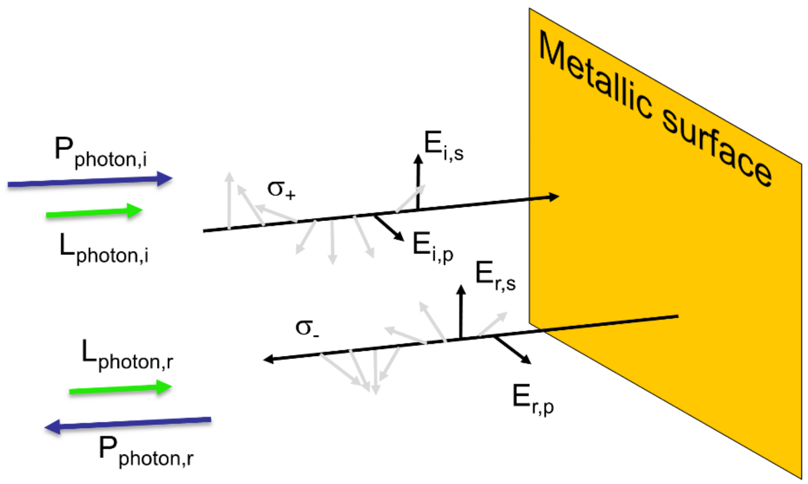

Fig. S5: Helicity change of the photon reflected from a metallic surface with incoming angle of 0°. The reflected photon changes from σ+ to σ- polarised or vice versa. $\mathbf{P}_{\mathbf{Photon}}$ and $\mathbf{L}_{\mathbf{photon}}$ depict the linear and angular momentum of the photon before (i: initial) and after (r: reflected) reflection of the surface. In can be seen from the sketch that while the linear momentum reverses and is transferred, and the helicity reverses aswell from σ+ to σ-, because of the reversed traveling direction, the momentum vector $\mathbf{L}_{\mathbf{photon}}$ of the angular momentum stays unchanged after reflection.

First, we calculated the number of photon reflected per atom within the penetration depth. We obtain the surprising result that for high fluencies, close of the destruction threshold of the material, the number of photons in the reflected light is, for the laser parameters of 7.5 mW at 250 kHz and photon energy of 1.55 eV, estimated to a laser energy per pulse of $30 \cdot 10^{-9}$ J which result in a number of photons per pulse of about $1.208 \cdot 10^{11}$. To compare this to the number of atoms in the focal volume we take the $L1_0$ crystal cell volume of $54.93 \cdot 10^{-30}$ m^3 and a 14 nm penetration depth and radius of 8.5 µm of the excitation spot. Then the number of atoms in the focal volume amounts to $1.156 \cdot 10^{11}$, which gives about *1.04 photons per atom*. Each is carrying an angular momentum of $\hbar$. This is different to the argumentation by *Dalla Longa et al.*, who calculated the number of absorbed photons to be negligible small for the low fluence range [7]. That means,

from the number of photons the transfer of an angular momentum from the photon in reflection could in principle be a dominating mechanism.

However, it was also shown that in reflection the effect is a little bit more complex. In case of an isotropic media, the polarisation state of the reflected light is determined by the angle of incidence. The component $\mathbf{E}_{r,p}$ can have a sign change thought boundary conditions imposed by Maxwell's equations which would be equal to a relative phase change of the components $\mathbf{E}_{r,p}$ and $\mathbf{E}_{r,s}$. For an incidence angle of 0°, the relative phase is not changed, and we obtain a reversed helicity upon reflection from σ+ to σ- or vice versa. However the angular momentum $\mathbf{L}_{photon}$ relative to the sample is not changed due to the, at the same time reversed, traveling direction of the light (Fig. S5). Indeed, it was shown that an angular momentum transfer by reflection from a perfect reflecting metallic isotropic film is not possible [8]. It is only possible in specifically shaped metamaterial resonators or ring structures [9].

As mentioned above, Beth showed that angular momentum of light could be transferred to a medium, leading to a tiny rotation of the medium. This was achieved for a *birefringent* medium and is thus not in contradiction to the zero effect for an isotropic medium. In particular, the birefringence condition on the dielectric tensor in Beth's experiment was $\varepsilon_{xx} - \varepsilon_{yy} \neq 0$, to have a nonzero effect. However, in our case the $L1_0$ FePt particles are isotropic in the basal plane, i.e. they have $\varepsilon_{xx} = \varepsilon_{yy}$.

In addition, it is important to note that the mechanism of Beth results in an electric torque on the electrons, which is transferred by coupling to the lattice to the whole sample, leading to a macroscopic rotation of the sample. Note that there is no direct torque exerted on the magnetisation. In order to influence the spin system a very efficient coupling would be needed from the lattice. It is however more likely that, for the here investigated sample, the torque on the sample is dissipated to the environment (substrate).

References:

[1] Weller, D., Mosendz, O., Parker, G., Pisana, S., Santos, T. $L1_0$ FePtX-Y media for heat-assisted magnetic recording, Phys. Status Solidi A **210**, 1245 (2013).

[2] El Hadri, M. S. *et al.* Electrical characterization of all-optical helicity-dependent switching in ferromagnetic Hall crosses, Appl. Phys. Lett. **108**, 092405 (2016).

[3] Takahashi, Y.K. *et al.* Accumulative magnetic switching of ultra-high-density recording media by circularly polarized light, Phys. Rev. Applied 6, 054004 (2016).

[4] Medapalli, R. et al. Mechanism of all-optical control of ferromagnetic multilayers with circularly polarized light, arXiv:1607.02505.

[5] Beth, R. A. Mechanical detection and measurement of the angular momentum of light, Phys. Rev. **50**, 115-125 (1936).

[6] Friese, M. E. J., Nieminen, T. A., Heckenberg, N. & Rubinsztein-Dunlop, H. Optical alignment and spinning of laser-trapped microscopic particles, Nature **394**, 348–350 (1998).

[7] F. Dalla Longa, J. T. Kohlhepp, W. J. M. de Jonge, and B. Koopmans Phys. Rev. B **75**, 224431 (2007).

[8] Mansuripur, M., Zakharian, A. R., & Wright, E. M. Spin and orbital angular momenta of light reflected from a cone, Phys. Rev. A **84**, 033813 (2011).
[9] Émilea, O., Brousseaub, C. Émilec, J., & Mahdjoubib, K. Energy and angular momentum transfers from an electromagnetic wave to a copper ring in the UHF band, C. R. Phys.18, 137–143 (2017).
